# Rare Complications of CSF Diversion: Paradoxical Neuroimaging Findings in a Double, Chiasmic Case Report

**DOI:** 10.3390/diagnostics14111141

**Published:** 2024-05-30

**Authors:** Gianfranco Di Salle, Gianmichele Migaleddu, Silvia Canovetti, Gaetano Liberti, Paolo Perrini, Mirco Cosottini

**Affiliations:** 1 Academic Radiology, Department of Translational Research, University of Pisa, Via Roma 67, 56126 Pisa, Italy; gianfri1810@gmail.com; 2 Neuroradiology Unit, Azienda Ospedaliero Universitaria Pisana, 56124 Pisa, Italy; s.canovetti@ao-pisa.toscana.it; 3 Neurosurgery Unit, Azienda Ospedaliero Universitaria Pisana, 56124 Pisa, Italy; 4 Department of Translational Research on New Technologies in Medicine and Surgery, University of Pisa, 56126 Pisa, Italy; 5 Neuroradiology Unit, Department of Translational Research on New Technologies in Medicine and Surgery, University of Pisa, 56126 Pisa, Italy

**Keywords:** cerebrospinal fluid, shunt, MRI, slit ventricle system, isolated dilation of the fourth ventricle

## Abstract

Two patients with CSF shunting systems exhibited symptoms of altered intracranial pressure. Initial neuroimaging led to misinterpretation, but integrating clinical history and follow-up imaging revealed the true diagnosis. In the first case, reduced ventricular size was mistaken for CSF overdrainage, while the actual problem was increased intracranial pressure, as seen in slit ventricle syndrome. In the second case, symptoms attributed to intracranial hypertension were due to CSF overdrainage causing tonsillar displacement and hydrocephalus. Adjusting the spinoperitoneal shunt pressure resolved symptoms and imaging abnormalities. These cases highlight the necessity of correlating clinical presentation with a deep understanding of CSF dynamics in shunt assessments.

Case 1

A 52-year-old female was referred to our neuroradiology division due to headache and visual impairment, with no response to medical treatment.

She was diagnosed with idiopathic intracranial hypertension (IIH) based on a highly suggestive clinical presentation (chronic and drug-resistant cephalgia and progressive visual loss with no alternative explanation). MRI, performed upon the patient’s admission (Figure 1), showed asymmetry of the transverse sinuses, suggesting incomplete stenosis (a), mild turgidity of the optic nerve sheath (b), and normal ventricles (c). Together, these findings strengthened the suspicion of IIH [1,2]; therefore, the patient was referred for neurosurgical treatment. Placement of a ventriculoperitoneal shunt (VPS) (Hakim Codman programmable valve, initial valve-opening pressure of 140 cmH_2_O) revealed intracranial hypertension and led to symptom relief. Normal CSF composition and the absence of localizing signs or intracranial masses confirmed the diagnosis of IIH according to modified Dandy’s criteria [3].

Five months after shunting, continuous and sharp cephalgia arose; MR scans revealed findings suggestive of intracranial hypotension, possibly due to overdrainage (Figure 2a–h). The patient was therefore admitted into hospital, and her shunt was equipped with an anti-siphon device. According to the initial suspicion of overshunting, the valve-opening pressure increased to 200 cmH_2_O, and eventually, the system was mechanically closed. Despite this, during the patient’s stay, she developed progressive visual impairments, aphasia, and motor incoordination in addition to her pre-existing symptoms, culminating in sensory obnubilation. A few weeks later, a further MR scan showed diffuse demyelination-like alterations of the deep white matter in both hemispheres, along with findings still suggestive of intracranial hypotension, such as collapsed ventricles, dural thickening at the vertex, small sulci, turgidity of dural venous sinuses and brain superficial veins (Figure 2i–p). Insertion of a catheter for ICP monitoring revealed marked intracranial hypertension (50 mmHg). A posteriori evaluation of MR images could not identify any secondary cause of intracranial hypertension. Direct causes of the observed visual impairments were excluded based on MRI examinations, which were persistently negative for optic pathway disease.

Since no MRI sign of intra-cranial hypertension was visible in this examination, the increased ICP found at the time of the new catheter insertion was surprising. The coexistence of collapsed appearance of the lateral ventricles and evidence of correct shunt functioning without overdrainage led to the diagnosis of slit ventricle syndrome. An emergency decompressive craniectomy could not prevent the patient’s death.

Case 2

A 23-year-old female patient had been suffering from headache.

At 5 months of age, she was diagnosed with obstructive hydrocephalus, which occurred as a complication of intraventricular hemorrhage. She was successfully treated with the placement of a VPS in the right lateral ventricle. At age 13, she underwent shunt revision due to malfunction, leading to its removal. In its place, a third ventriculocisternostomy (TVC) was performed, followed by the placement of a spinoperitoneal shunt (SPS), which is still in place today.

Two years before being referred to our hospital, the patient developed a progressive orthostatic headache combined with mental confusion and lower limb hyposthenia that partially recovered, assuming a supine position. An MR examination revealed isolated dilatation of the fourth ventricle and supposing intracranial hypertension related to IV ventricle hydrocephalus; further ventricular shunting was proposed.

Upon her admission to our hospital, an MRI exam was repeated with contrast media administration. On T2WI, the fourth ventricle was markedly enlarged (Figure 3a and Figure 4a), compressing the pons anteriorly and the cerebellum posteriorly. Accordingly, subtentorial cisterns were effaced. A thin diaphragm was appreciable within the acqueductus Silyii. Axial scans of the enlarged fourth ventricle showed patent and enlarged foramina of Luschka (Figure 3a). T2W hyperintensities in the ventricle walls and around the cranial portion of the central canal suggested trans-ependymal reabsorption and incipient hydromyelia (Figure 4b,c). The MRI exam showed caudal dislocation of the bulbo-medullary junction and herniation of cerebellar tonsils through the foramen magnum. On the other hand, T1W C+ scans showed a diffuse enhancement of the thickened dura mater, surrounded by hyperintense epidural spaces (Figure 4b).

The patient was then diagnosed with isolated dilatation of the fourth ventricle, caused by overdrainage from the SPS causing downward tonsillar displacement as the origin of IV ventricle intracranial and spinal hydrocephalus. The increase in the SPS valve-opening pressure, with subsequent downregulation of the shunt, led to prompt symptom regression and the disappearance of hydromyelia and other MRI signs of trans-ependymal reabsorption (Figure 4e,f).

Intracranial pressure (ICP) and cerebrospinal fluid (CSF) dynamics are tightly interconnected. Monro–Kellie doctrine, until its most recent reformulations [4], states that ICP is kept constant by reciprocal and opposite volume changes in brain compartments, namely nervous tissue, blood vessels, and CSF, with possible adaptations of the skull. CSF volume can adapt rapidly to acute and subacute modifications in other brain compartments; on the contrary, counterbalance to CSF flow dynamics alterations by venous compartment is limited. For this reason, altered CSF dynamics are often accompanied by ICP variations, which in turn can have a serious clinical impact. For the same reason, CSF shunts can serves as both therapy and a cause of ICP imbalances. In the United States alone, CSF shunts are placed in several tens of thousands of patients every year [5]. Despite their leading role in common neurosurgical practice, their management is extraordinarily costly, with an average cost of over USD 35.000 per patient. This is because complications are common, as they affect up to 40–50% of patients in the first two years, often requiring surgical revision (22% in 4 years) [6]. Recent literature data about CSF shunt complications focus on shunt obstruction, infection, overdrainage, mechanical failure, and catheter site-specific failures [7]. Yet, the correct functioning of a shunt also depends on the assumptions made on the underlying anatomy of CSF spaces. On the one hand, intracranial CSF spaces are not rigid, as they comply with volume variations; on the other hand, ICP variations induced by the shunt can in turn modify CSF spaces anatomy, with consequences for flow dynamics and shunt effects on the pressure balance. Therefore, complications of CSF shunting are not limited to those listed previously, but shunting effects can in turn modify CSF space anatomy and physiology and create a new balance, in the light of which clinical evaluation and shunt regulation/revision should be performed. In other words, surgical management of shunt complications should always be based on a thorough clinical evaluation and a profound knowledge of CSF dynamics.

In the two cases presented above, shunting modifies local anatomy and CSF dynamics, leading to opposite, paradoxical neuroimaging findings. The key to conundrum resolution will only be obtained by correctly deciphering the combination of clinical presentation and imaging findings.

***Slit ventricle syndrome*** (SVS) is a complication in patients with hydrocephalus who have undergone CSF drainage, occurring in 4–37% of patients who undergo shunt surgery [8]. This condition has been defined as “a syndrome of intractable headaches in shunted patients with small ventricles” [8]. Despite its pathogenesis being poorly understood, it has been postulated that a combination of venous congestion and sub-ependymal gliosis could reduce ventricle wall compliance, thus preventing an appropriate dimensional increase in the ventricles with coexisting intracranial hypertension [9]. For this reason, in the past few years, this condition has become known as “non-compliant ventricle syndrome”.

Pathophysiological explanations of this condition encompass concurrent mechanisms, such as shunt overdrainage, compensatory venous congestion (as we documented in our case, Figure 2f–h), and altered compliance of the ventricular system and cerebral tissue [8]. In patients with shunt-associated headache, therapeutic management is traditionally based on migraine medication [10] and diuretics [11]. Refractory cases were classified by Panagopoulos et al. [8] into two categories: headache due to intracranial hypotension (overdrainage) and pathological compliance of the ventricular system (classical SVS). In our case, ambiguous MRI findings led to the misclassification of patients in the former category when they actually belonged to the latter. Reduced ventricular compliance masked the diagnosis of intracranial hypertension and favored the misdiagnosis of hypotension. The correct diagnosis of SVS led us to reactivate the shunting device and, eventually, perform a decompressive craniectomy, but this was not timely enough to stop the symptom progression. Notably, the physiology of CSF intra- and extra-ventricular circulation was preserved. Orthodromic circulation was present from the choroid plexus in the ventricles to the arachnoid granulations, without obstructive phenomena at any stage. CSF shunting was necessary to counteract intracranial pressure increase, worsened by reduced ventricular wall compliance. The treatments we performed included all of the widely accepted recommendations for headache due to intracranial hypotension, namely: (1) the addition of an anti-siphon device; (2) an increase in valve opening pressure; (3) a combination of both in refractory cases; and (4) careful check of the ventricular catheter and replacement in cases of obstruction [8]. Treatment of proper SVS, with pathological compliance of the ventricular system, is more controversial. Proposed interventions are highly heterogeneous and include shunt revision/repositioning/substitution, third ventriculostomy, an increase in valve opening pressure, and decompressive surgery [8,12]. This is because appropriate treatment should be provided according to the underlying pathophysiological context, not the epiphenomenon of altered ventricular compliance. Endoscopic third ventriculostomy can be appropriate in cases of raised ICP and obstructive hydrocephalus; shunt revision when obstruction is exacerbating ICP increase in combination with altered ventricular compliance; and increase in valve opening pressure in shunted children where dampened CSF pressure waves have induced abnormal calvarial synostosis [13]. Further research is needed to clarify the emerging role of the glymphatic system in IIH pathogenesis [14,15] in order to propose less invasive and disease-modifying treatments. To the best of our knowledge, no medical treatment targeting aquaporin-4 changes or neurogliovascular unit disruption in IIH has ever been developed, currently leaving no alternatives to surgical treatments—and complications.

The variety of available treatments for SVS, as well as the complexity of the clinical case we have described, together point to the need for a profound comprehension of the clinical context when choosing the most appropriate management of SVS [8,12].

***Isolated dilatation of the fourth ventricle*** is a rare complication of ventriculoperitoneal shunt surgery, with it being most frequent among pediatric patients and poorly characterized in adults [16,17]. Necessary conditions are, typically, obstruction of the foramina of Luschka and Magendie, inferiorly, and the aqueduct of Sylvius, superiorly. In our case, congenital aqueductal stenosis was present (Figure 4a,d), but foramina were apparently patent—even symmetrically dilated (Figure 3a), while the site of obstruction was farther below. SPS overdrainage caused brainstem and tonsillar sagging, foramen magnum obstruction, and CSF flow trapping in the posterior fossa. Thus, the central canal became the only outflow route available, communicating with the high-pressure closed compartment of the fourth ventricle. Below the foramen magnum, a radial pressure gradient was present from the central canal (abnormally increased pressure) to the spinal subarachnoid space (abnormally decreased pressure). Conspicuous trans-ependymal reabsorption was then provoked in the spinal cord (Figure 4c), at risk of further extension with involvement of the brainstem. The therapeutic approach consisted of only modifying the drainage pressure of the shunt, thus increasing peri-medullary space pressure to normal levels and removing the CSF trapping in the posterior fossa. In turn, this reduced the pressure in the fourth ventricle and removed the gradient established between the central canal and the medullary sub-arachnoid space. The patient’s symptoms rapidly alleviated, without more invasive interventions being required. In subsequent MR examinations, further ascent of cerebellar tonsils was observed, with further resolution of fourth ventricle isolation. Nevertheless, long-term follow-up is needed to monitor the occurrence of late complications, including relapse of overdrainage with spinal subarachnoid space compartmentalization.

In these two cases, we show conflicting evidence coming from clinical presentation and gross morphological examination through neuroimaging. The first patient presented with symptoms and MRI results suggestive of intracranial hypotension, but she was actually affected by slit ventricle syndrome, masking intracranial hypertension. On the other hand, the second patient presented with hypotension symptoms and paradoxical fourth ventricular hydrocephalus requiring urgent therapeutic intervention because of a high-pressure intraventricular and central canal functional compartment.

In conclusion, patients treated with a CSF shunt can undergo radical anatomic and functional variations, such that the first interpretation of the diagnostic images can be misleading. Clinical correlation and a profound comprehension of CSF flow dynamics are therefore mandatory to make the correct diagnosis and provide the correct treatment.

## Figures and Tables

**Figure 1:**
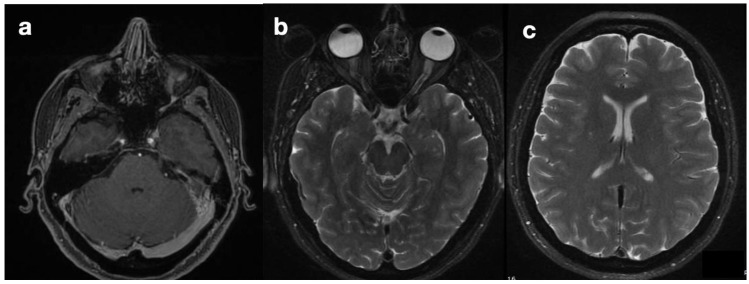
Preoperative MRI. Images showing the findings of the asymmetry of transverse sinuses (**a**), optic nerve sheath enlargement (**b**), and normal ventricles (**c**), compatible with intra-cranial hypertension.

**Figure 2:**
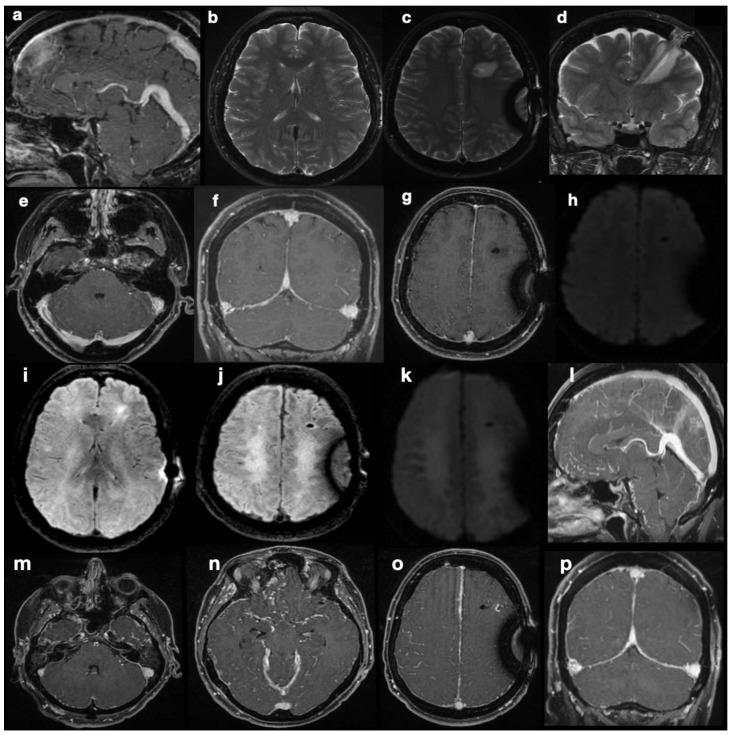
Post-shunting MRI scans. The first post-operative MRI (**a**–**h**) is highly suggestive of intracranial hypotension, with “slumping midbrain” and depression of the vena magna Galeni (**a**), small ventricles (**b**), and supra- and sub-tentorial dural thickening with turgidity of the venous sinuses (**f**–**h**). At the same time, a fluid effusion surrounds the parenchymal course of the catheter, hinting at draining dysfunction. DWI sequences did not show abnormal findings (**e**). MR scan after marked neurological deterioration (**i**–**p**) shows ongoing demyelination of deep periventricular white matter in both hemispheres (**i**–**k**). Radiological signs suggestive of intra-cranial hypotension are even more conspicuous here than previously described in images (**a**–**h**).

**Figure 3:**
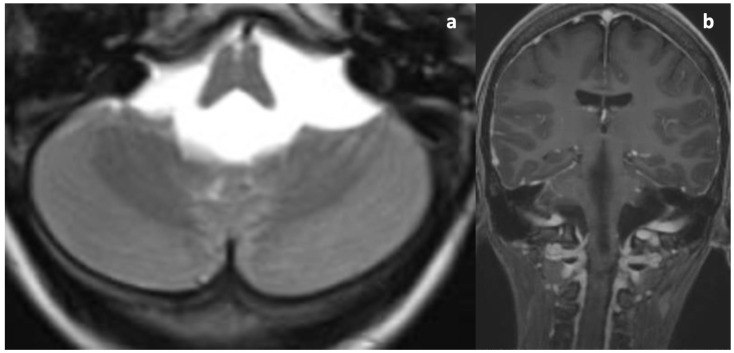
T2WI and C+T1WI upon admission. (**a**) Symmetrical dilatation of the foramina of Luschka. No cystic malformation or space-occupying lesion can be seen. Ventricular outlets are pervious (patient in a lying position). (**b**) Thickening and contrast enhancement of the dura mater; note the normal dimensions of the lateral ventricles due to the normal functioning of the TVC.

**Figure 4:**
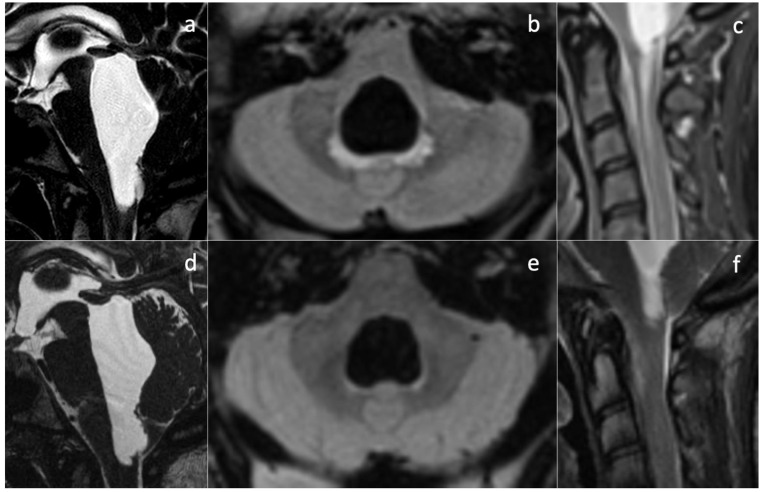
Effect of SPD revision on imaging findings. Isolated dilatation of the fourth ventricle is visible before (**a**) and after drainage downregulation (**d**). Partial re-expansion of subtentorial cisternal spaces can be seen. A subtle membrane is responsible for the obstruction of the cerebral aqueduct. Signs of peri-ventricular trans-ependymal reabsorption (**b**) and hydromyelia (**c**) disappear after shunt revision ((**e**,**f**), respectively).
